# Supplementary material for: Are carbon emissions trading and green financial instruments synergistic? -Comprehensive quantitative research based on content analysis
Source: PLoS One. 2024 Mar 7;19(3):e0298601. doi: 10.1371/journal.pone.0298601 (PMC10919601; doi:10.1371/journal.pone.0298601)
Supplement: S1 Data — (ZIP) [file pone.0298601.s001.zip › Data/地级市Z_F工作报告.zip!/║■─╧/╘└╤⌠╩╨/└┤╫╘╣1⁄2╓┌║┼í╛┬φ┐╦╩2╛▌═°í┐_2019.docx]

各位代表：

现在，我代表市人民政府向大会报告工作，请予审议，并请市政协委员和其他列席人员提出意见。

一、站在新时代历史起点，岳阳高质量发展迈出坚实步伐

2018年，在中共岳阳市委坚强领导下，在市人大、市政协监督支持下，我们坚持以习近平新时代中国特色社会主义思想为指导，全面贯彻党中央、国务院和省委、省政府决策部署，积极践行“一三五”基本思路，大力推行一线工作法，强力推动岳阳高质量发展，加快建设湖南发展新增长极，全市GDP增长8.3%，规模工业增加值、固定资产投资、社会消费品零售总额、一般公共预算收入分别增长7.6%、10.6%、9.3%和6.6%，经济社会发展稳进向好。岳阳成功获得“中国百强城市”“中国可持续发展百强城市”“中国外贸百强城市”“中国城市品牌”全国地级市第15强、“中国最具幸福感城市”第24强等城市名片，特别是中央备案同意《湖南省关于市县机构改革的总体意见》，将岳阳列入湖南省大城市行列，极大提振了全市人民的荣誉感和自豪感。

经济质量效益持续提升。坚持以提高质量效益为中心，经济发展“稳”的态势更加巩固、“进”的走势更加强劲、“好”的趋势更加凸显，三次产业结构由9.7：43.1：47.2调整为9.4：41.7：48.9。从经济板块看，产业园区越来越强。严格落实“园区十条”，积极探索“飞地经济”模式，既为产业园区发展减压松绑，又为产业同质集聚创造条件，园区规模工业增加值占比达到81.9%，同比提高7.3个百分点，工业实缴税金增长15.8%，产业园区成为岳阳高质量发展的强大引擎。从经济结构看，非公占比越来越大。首次召开企业家大会，广泛凝聚了“服务企业就是服务发展”“尊重企业家就是尊重生产力”的社会共识。严格落实“非公十条”，组建政府性融资担保公司和小微企业信贷风险补偿基金，扶持发展非公经济，各项贷款余额增长24.8%，居全省第一位；新入库“四上”单位693家，居全省第二位；新登记各类市场主体4.79万户，总量居全省第二位；非公经济占GDP比重达到65.5%。从经济效益看，财税质量越来越高。严格防控政府债务风险，停缓调撤政府投资项目162个，调减金额713.3亿元，编制实施隐性债务10年化解方案，政府债务风险总体可控；市属国有企业整体扭亏为盈；税收收入占地方收入比重同比提高11.9个百分点。

产业项目建设持续发力。强力推进“产业项目建设年”活动，我市产业项目建设走在全省前列。产业招商刷新了“成绩单”。组织开展“产业招商月”活动，引进投资过100亿元工业项目2个、10—50亿元工业项目23个、5—10亿元工业项目20个，其中签约引进“500强”工业企业16家，竣工投产“500强”工业项目4个。特别是总投资208.6亿元的己内酰胺搬迁扩能升级项目，将推动我市加快打造2000亿石化产业集群；总投资过100亿元的新金宝年产1300万台喷墨打印机项目，将推动我市加快打造1000亿电子信息产业集群。项目建设跑出了“加速度”。实施重大项目开工竣工“双考核”、市级领导一对一盯项目、重大产业项目市级层面直接调度等制度，形成了产业项目建设长效机制。省定“五个100”项目强力推进，38个项目有34个成功落地、20名专家有19名签约到岗。市定“双百工程”扎实推进，100个重大前期项目81个达到预期进度，105个重大建设项目完成投资459.8亿元；63个重大产业项目在建和竣工36个；33个市级领导联系的重大产业项目在建和竣工19个；复星合力新材料从签约到开工仅用2个月时间，哈工高端液压测试装备、海正生物酶制剂等19个亿元项目实现当年开工、当年竣工。技改扩能按下了“快进键”。坚持对外招商与对内挖潜“两手抓”、引进增量与盘活存量“两不误”，工业技改投资增长64.7%，科伦制药、华文食品、新金刚等一批企业扩能倍增，产值税收实现“两个翻番”。过去一年，我市产业招商培育了一批经济增长点，项目建设形成了一批实物工程量，技改扩能催生了一批现实生产力，实体经济发展好戏连台、捷报频传，岳阳现代产业新格局加快落棋布子、加快升级成型！

对外开放发展持续升温。深入实施开放崛起战略，强力推进港口建设大会战，在长江中游地级港口城市中，我市开放型经济发展正由“跟跑者”向“领跑者”转变。开放指标高速增长。完成进出口总额31.2亿美元，增长37.3%；集装箱吞吐量达到66.8万标箱，总量大幅超过九江和宜昌；岳阳综保区进出口总额稳居全省7个海关特殊监管区第一位。进口粮食、肉类总量均实现翻番，平行进口汽车突破5000台，居全国26个同类口岸第5位。开放平台继续拓展。推动组建湖南省港务集团，岳阳航运交易中心、国际贸易“单一窗口”、外贸联检锚地等建成运营，新港二期、综保区二期、跨境电商平台等扎实推进。坚持“南客北货”定位，出台空港经济区发展规划，三荷机场建成通航，岳阳正式进入航空时代。开放主体逐步增多。鼓励支持外贸企业开拓国际市场，245家外贸企业产生业绩，同比增加39家，114家企业“破零倍增”，外向型经济市场主体争相涌现、竞相发展。开放政策全面落地。推动省委、省政府支持岳阳开放发展的各项政策落实见效，设立现代物流产业发展基金，严格兑现“开放十条”，累计奖励开放型企业1.2亿元。国际商贸保税物流中心建成运营，空港物流园、临港汽车城物流园、海吉星农产品物流园等加快建设，A级以上物流企业达到33家，居全省第二位，岳阳被列为全国30个港口型物流枢纽城市之一，正朝着千亿现代物流业城市阔步前进。同时，商贸、金融、旅游等现代服务业蓬勃发展，友阿国际广场等城市商业综合体加快建设；成功引进平安银行、广发银行；圆满举办洞庭湖观鸟节、中国（湖南）红色旅游文化节等活动，岳阳楼—君山岛景区荣登全国5A级景区口碑红榜前三甲，临湘获评中国健康养生休闲度假旅游最佳目的地，汨罗西长村获评中国美丽休闲乡村，现代服务业对经济增长贡献率提高到51.5%。

乡村振兴战略持续推进。按照中央“20字”总要求，突出抓好“五个振兴”，成立岳阳乡村发展研究中心，积极探索乡村振兴新路径，开启了“三农”工作新局面。“空心房”整治“小改革”打造了“大亮点”。确定52个重点乡镇先行先试，拆除“空心房”1089万平方米，腾退土地7.7万亩，复耕交易耕地指标2.47万亩，整治复绿6832亩，我市“空心房”整治新模式获评“改革开放40年地方改革创新40案例”。规范村民建房“小切口”做出了“大文章”。编制村庄规划357个，规划集中建房点300个，累计建成253个，农村建房审批率100%，节约用地9800多亩，1.5万农户住上梦寐以求的“小洋楼”。特色小镇创建“小产业”形成了“大品牌”。配合出台创建农业产业化特色小镇工作方案，选定20个乡镇启动示范创建，培育了岳阳黄茶、岳阳小龙虾、华容芥菜、平江豆干等一批特色产业，成功举办湖南岳阳小龙虾产业博览会、全国黄茶斗茶大赛暨岳阳茶业博览会、中国（华容）芥菜大会、第二届中国（平江）休闲食品文化节、“全能王”全国（临湘）钓鱼锦标赛，地域特色产业知名度明显提高。全市国家地理标志保护产品达到11个，居全省第一位；规模农产品加工业产值达1622.7亿元，增长10.4%。农村移风易俗“小整治”淳化了“大风气”。持续开展“刹人情歪风、治婚丧陋习、树文明新风”专项行动，严肃整治人情“通知单”异化为“催款单”的不良陋习，1824个村（社区）实现全覆盖，农村“人情债”更少了，“人情味”更浓了。

生态环境治理持续攻坚。牢记习近平总书记“守护好一江碧水”的殷切嘱托，把保护长江生态环境摆在压倒性位置，坚决做到守土有责、守土尽责。坚持高标准谋划。全面贯彻习近平总书记在深入推动长江经济带发展座谈会上的重要讲话精神，统筹推进工业排污整治等“七大行动”，率先全省推行河长制“五个一”工作机制，“绿色岳阳”建设迈出坚实步伐。坚持高强度推动。中央环保督察6项未销号问题完成年度整改任务，下塞湖矮围整治得到国务院联合调查组充分肯定，绿色化工产业园云溪片区环境问题整改任务基本完成，河道采砂治理、排污排渍口整治、饮用水源地保护、河湖“清四乱”等工作走在全省前列，洞庭湖生态环境整治取得阶段性成效。突出长江岸线码头整治，拆除小散泊位42个，关停危险渡口13道，退出长江岸线7.3公里，得到中央第四环保督察组高度肯定；突出黑臭水体整治，27处年度任务基本完成，中心城区最大的黑臭水体东风湖加快重现水清岸绿“新画卷”。坚持高规格协作。加强与三峡集团合作，南港河、北港河综合整治等一批先行先试项目落地实施。过去一年，广大干部群众克服重重困难，排除种种阻力，以背水一战的勇气、壮士断腕的决心，强力推进污染防治攻坚，岳阳青山常在、绿水长流、蓝天永驻的生态发展新格局正在加快形成！

基础设施体系持续改善。坚持基础先行，着力扩大有效投资。交通方面：继续实施交通建设大会战，蒙华铁路岳阳段进展顺利；岳望高速建成通车，平益高速、城陵矶高速等扎实推进，高速公路通车里程近500公里，实现了“市区10分钟上高速、县县通高速”目标；湘北大道、长康南路、奇康路、汴河园路等7个限时竣工项目建成通车，东环线、南环线全面打通，北环线加快建设，一批交通“节点”逐个打通。水利方面：扎实推进洞庭湖北部地区补水工程、南湖电排站扩容改造、黄盖湖综合治理、梅溪港河治理、黄梅港河修复和蓄洪安全区建设，除险加固病险水库101座，清淤大中型渠道1417公里，建成高效节水灌溉面积2.6万亩，兴建农饮项目180处，解决了58.3万农村人口饮水不安全问题，水利防汛保安、生态涵养、农田灌溉、安全饮水四大保障能力全面提高。能源方面：华电平江电厂、平江抽水蓄能电站等实现重大推进，新建和改造110千伏变电站7个，完成行政村配电网改造154个。信息方面：新建光缆管道220余公里，4G覆盖率达99.26%。基础设施承载能力明显增强，硬件基础成为岳阳高质量发展的硬性支撑。

改革创新红利持续释放。坚持以供给侧结构性改革为主线，打出了一套改革创新的“组合拳”。推进重点领域改革。深化“放管服”改革，配合出台“最多跑一次”改革实施方案和服务企业优化环境“九条措施”，确定“最多跑一次”事项1096项，占依申请类事项94.2%；市政务服务大厅进厅事项新增537项，达到707项；率先全省完成“互联网+政务服务”改革任务，实现“最多跑一次、最好不要跑、最长一百天”的阶段性目标。积极创建全省深化供销合作社综合改革示范市，市县供销社独资和参股企业达到156家，购销额增长233%，率先全省探索了可推广、可复制的经验。深入推进屈原农村改革集中区建设，为破解“三农”难题探索了新路子。同时，统筹推进了机构改革、财税改革、农村综合改革、商事制度改革等各项改革。实施创新引领战略。全社会研发投入增长24.1%，新增高新技术企业78家，增长40.6%，高新技术产业增加值占GDP比重达20.6%，湘阴获评全国首批创新型县，哈工特飞荣获湖南创新创业大赛一等奖。深入实施巴陵人才工程，推出创业扶持、创新资助、引智补贴、政府奖励、配套服务、绿色通道6大扶持政策，引进柔性挂职专家117名、博士以上人才30名，岳阳高质量发展的机制活力、创新动力加快释放。

人民生活水平持续提高。坚持为民理财用财，财政民生支出占比超过70%，居民人均可支配收入增长9%。突出抓好脱贫攻坚。深入推进脱贫攻坚大会战，109个贫困村出列，6.06万人成功脱贫，综合贫困发生率下降至0.91%，平江县“摘帽”通过省级考核评估。全市投入扶贫资金4.5亿元，整合涉农资金7.3亿元，完成易地搬迁安置1.77万人，纳入低保兜底保障对象1.04万人，贫困村种养产业村级覆盖率达100%，就业、教育、金融、健康扶贫等政策全面落地。突出抓好“两改”工作。完成省定棚改任务3.2万户、农村危改任务1.44万户。“十大片区”棚改项目、55个棚改项目和“五个5”惠民工程强力推进，“清零”项目30个，征拆房屋291.4万平方米，搬迁20189户，腾地6325亩；开工建设定向限价商品房4100套，“限房价、竞地价”效果明显。突出抓好社会事业发展。中小学校“六建六扩”顺利推进，白杨坡小学、市十九中投入使用，化解“大班额”总量居全省第一；医疗资源布局调整强力推进，组建市一医院医疗集团，建成医疗联合体128个，岳阳获评全国无偿献血先进市，造血干细胞捐献居全国地级市第一位；文化惠民工程扎实推进，文化产业快速发展；成功举办首届“健康湖南”全民运动会、“屈原杯”汨罗江国际龙舟拉力赛，我市运动员斩获3枚亚运会金牌，创历史最好成绩。突出抓好实事办理。省、市22件实事基本完成。新增城镇就业5.3万人；开展中心城区交通秩序综合整治，推行公安、城管和交通联合执法，新增公共停车位967个，新建人行过街设施3处，拉通“盲肠路”11条；建成自然村通水泥（沥青）路1698公里，临湘获评“四好农村路”全国示范县；珍珠山公园基本建成；中心城区五个“一公里公共服务圈”基本形成；57家临街单位向社会免费开放共享厕所，39家机关单位对外免费开放内部停车场；食品药品集中整治成效明显；基本社会保险覆盖率达99.4%，企业退休职工基本养老金和城乡低保标准每月分别提高到2167元和480元、285元，岳阳获批全国居家和社区养老试点城市。突出抓好社会治理。扎实开展扫黑除恶专项斗争，打掉了一批涉黑涉恶犯罪团伙，刑事发案率下降21.3%，平安创建深入人心，岳阳楼区成功创建全国社区治理和服务创新试验区；森林火灾受害率下降86%，各类安全事故下降57.1%，岳阳获评全国“安全生产月”活动先进单位；信访“三无”单位创建工作走在全省前列；我市顺利通过全国文明城市复查测评，屈原义南村获评“全国文明村”。

民主法治建设持续加强。全面推进依法行政，实施政府公职人员宪法宣誓，彰显了宪法的权威性和宣誓的仪式感；自觉接受人大法律监督、工作监督和政协民主监督，承办人大代表建议和政协提案523件，办结率、满意率分别达100%和99.8%；配合市人大出台《岳阳市东洞庭湖国家级自然保护区条例》《岳阳市机动车停车管理条例》等地方法规，制定实施《岳阳市政府合同管理办法》等政府规章，我市生态法治建设项目获第五届“中国法治政府奖”提名奖。

加强政府自身建设，常态开展专题学习教育，将政治理论、法律法规学习列入市政府常务会第一议题，主动“充电补能”；严格落实党风廉政建设“一岗双责”，加强行政监督和审计监督，出台实施《关于加强市政府系统党风廉政建设着力建设廉洁政府的意见》，为打造廉洁政府设定路标；深入践行一线工作法，实干实为、清正清廉成为广大党员干部的一种行动自觉和职业操守。

其他事业繁荣发展，国防动员、武警、消防、人防等有了新进步；老干老龄、史志、档案、国安、保密、公积金、科协、气象、水文、民宗、烟草、石油、盐业、邮政、通讯、联络接待等有了新发展；工会、共青团、妇联、侨联、残联、台办、贸促、工商联及各民主党派、无党派人士等作出了新贡献。

各位代表，前进的道路无比艰辛，岳阳的跨越发展却势不可挡！一年来的艰难险阻，没有消减全市上下干事创业的豪迈激情；一年来的砥砺奋进，更加增强了全市上下奋勇争先的高昂斗志；一年来的发展成果，再次提振了全市上下跨越赶超的必胜信心。我们深刻体会到：只有始终站稳政治立场，才能坚守岳阳发展的正确方向，一年来，我们坚定不移贯彻习近平新时代中国特色社会主义思想，以坚贞不渝的政治态度、坚如磐石的政治定力，推动政府工作沿着正确的轨道不断前行；只有认真践行基本思路，才能找准岳阳发展的现实路径，一年来，我们坚定不移落实市委提出的“一三五”基本思路，坚持谋大谋深谋远，全面落地落小落细，推动政府工作纲举目张、精准发力；只有勇于担当历史使命，才能激发岳阳发展的内生动力，一年来，我们坚定不移担负建设湖南发展新增长极的历史使命，面对艰难的外部环境和挑战，积极放大优势，努力补齐短板，与几大家班子一道协力同心、克难奋进，推动政府工作交出了一份难能可贵的“答卷”；只有紧紧依靠全市人民，才能凝聚岳阳发展的磅礴力量，一年来，我们坚定不移践行“发展为了人民、发展依靠人民”的工作理念，把群众呼声作为政府工作“风向标”，强化人民主人翁意识，发挥人民主力军作用，推动政府工作更多地增进了民生福祉、赢得了群众认同。

各位代表，过去一年，全市上下以“等不起”的紧迫感、“慢不得”的危机感、“坐不住”的责任感，撸起袖子加油干，一些特色工作走在全省前列。我市产业项目建设、农业产业化特色小镇创建、农村“空心房”整治、农村人居环境改善、供销社综合改革等一系列极具代表性、开创性、示范性工作经验在全省推介，说明省委、省政府对岳阳工作是充分肯定的。这些成绩的取得是市委坚强领导的结果，是市人大、市政协监督支持的结果，饱含了各位代表、各位委员的智慧和汗水，凝聚了全市人民特别是广大企业家、基层干部与各界群众的心血和力量。在此，我代表市人民政府，向全市人民，向所有关心、支持和参与岳阳改革发展的各界人士，表示衷心的感谢和崇高的敬意！

各位代表，过去一年，岳阳的发展确实特别艰难、异常艰辛，也让我们更清醒、更深切地认识到当前面临的困难和问题：一是发展的质量效益还需要进一步提高，主要是产业结构不优、财政质量不高、民营经济和县域经济实力不强，各类短板制约仍然较多；二是发展动能转换还需要进一步加快，主要是传统产业转型升级任务艰巨、新兴产业占比偏低、战略性产业项目总量偏少，新增长极建设任重道远；三是三大攻坚还需要进一步抓实，主要是隐性债务化解、生态环境治理、深度贫困户脱贫任务重、时间紧，必须久久为功抓落实；四是民生福祉还需要进一步改善，主要是城乡就业、教育医疗、社会保障、公益设施等与群众期望有差距，城乡发展仍不平衡；五是作风建设还需要进一步加强，主要是少数干部面对急难险重任务不想为、不会为、不敢为，导致部分工作推而不动，等等。对此，我们一定高度重视、认真解决。

二、认清新时代基本形势，始终坚定新增长极建设必胜信心

建设湖南发展新增长极，既是省委、省政府对岳阳发展的殷切期望，也是全市人民的共同梦想，需要我们一年接着一年干、一锤接着一锤敲，奋力推动美好梦想变成美丽现实。

推进新增长极建设，必须深入研判发展形势。当前，岳阳发展挑战与机遇并存，压力与动力同在。我们面临着前所未有的压力。主要是：国际贸易保护主义和单边主义抬头，我市外向型经济发展不确定、不稳定性因素明显增加；中央对生态保护高度重视、重拳出击，我市环保欠账多、基础弱，打赢污染防治攻坚战还需自我加压、持续发力；中央加强金融风险特别是政府债务风险防控，我市防风险的压力和扩投资的刚需“剪刀差”越来越大；区域竞争日益激烈，“标兵”遥遥领先，“追兵”步步紧逼，巩固全省“第二经济强市”地位需要经受新的考验。我们也面临着前所未有的机遇。主要是：中央将岳阳确定为区域性中心城市，正式备案赋予岳阳大城市地位，将助推我们加快构建大岳阳城市圈，增强岳阳对优质生产要素的吸附能力、对周边地区的辐射能力；中央强调做好“六稳”工作，聚焦实体经济、乡村振兴、生态保护和扩大有效投资等，采取了一系列重大政策措施，将推动我们加快构建“五个新格局”，增强岳阳高质量发展的动能和支撑；国家推进长江经济带、洞庭湖生态经济区发展战略，将倒逼我们转变发展方式、优化发展路径，增强岳阳生态优先绿色发展的底气和信心；省委、省政府将岳阳定位为“一极四区”城市，对岳阳发展给予强力支持，将激励我们做强“极”的实力、放大“区”的优势，增强岳阳跨越赶超、争先创优的信念和决心。当前，岳阳发展正处在爬坡过坎的转型期、厚积薄发的窗口期、大有可为的机遇期，全市上下要提振精气神，跑好接力赛，在建设富饶美丽幸福新湖南的宏伟征程中，建树新的业绩，作出新的贡献。

推进新增长极建设，必须全面理清发展思路。2019年政府工作总体要求是：坚持以习近平新时代中国特色社会主义思想为指导，全面贯彻党的十九大精神和中央、省委、市委经济工作会议精神，深入践行岳阳发展“一三五”基本思路，突出质量优先和绿色发展，进一步稳就业、稳金融、稳外贸、稳外资、稳投资、稳预期，继续打好“三大攻坚战”，大力实施创新引领开放崛起战略，加快推进国家区域性中心城市和湖南省大城市建设，加快打造湖南发展新增长极和长江经济带绿色发展先行区、大美洞庭湖生态环境保护区、内陆地区开放崛起示范区、中部地区现代经济体系建设试验区，为全面建成小康社会收官打下决定性基础，奋力书写新时代岳阳高质量发展的崭新篇章！

推进新增长极建设，必须科学确定发展目标。根据中央、省高质量发展的评价指标体系要求，建议今年主要预期目标分四个层面确定。主要经济指标：地区生产总值增长8%左右，规模工业增加值增长7.5%，固定资产投资增长11%，社会消费品零售总额增长10%，一般公共预算地方收入增长4%。创新发展指标：全社会研发投入增长22%，高新技术产业增加值占GDP比重达到23%，万人有效发明专利拥有量增长20%。生态环境指标：空气质量优良率达到85%，重要水功能区水质达标率达到95%，万元GDP能耗下降3.66%。民生保障指标：财政民生支出占比稳定在70%以上，居民人均可支配收入增长8.5%，居民消费价格指数涨幅控制在3%以内，城镇调查失业率控制在5%以内。

推进新增长极建设，必须精准把握发展要求。我们将一如既往尊重人民意愿、尊重经济规律、尊重地方实际，在事关方向、事关大局、事关长远的问题上保持定力、绝不动摇。坚持以人民为中心不动摇。政府是人民的政府，财政是人民的财政。我们将把保障改善民生作为政府工作的出发点和落脚点，无论财政压力有多大，都坚决做到民生投入只增不减、民生支出只升不降，主动向群众“晒账单”，把政府工作“点评权”“打分权”交给群众。坚持高质量发展不动摇。没有速度，发展就会停滞不前；没有质量，发展就会不可持续。我们将统筹稳增长与调结构平衡，实行扩总量与提质量并举，推动经济工作重心由“快不快”向“好不好”、“大不大”向“强不强”转变，持续推动岳阳高质量发展行稳致远。坚持生态优先绿色发展不动摇。生态环境治理“治顽症”更需要“下猛药”。我们将站在对历史负责、对人民负责、对岳阳负责的高度，主动适应环保工作新要求，坚定不移地推进产业生态化、生态产业化，坚决“守护好一江碧水”。坚持培育壮大民营经济不动摇。民营经济是推动发展不可或缺的力量，决不能弱化，绝不能“离场”。我们将毫不动摇鼓励、支持、引导民营经济发展，打造更加公平的市场环境、创造更加广阔的市场空间，让民营经济创新源泉充分涌流、创造活力充分迸发。坚持践行一线工作法不动摇。作风建设是事业成败的“生命线”。我们将腾出更多时间抓落实，集中更多精力解难题，主动到项目去、到企业去、到基层去、到现场去，用硬指标交账，用硬成绩说话，凭实干推动岳阳发展，凭实绩检验政府工作。

三、谱写新时代宏伟篇章，加快构建岳阳发展新格局

构建“五个新格局”是岳阳发展的新蓝图、新方略，我们要通过拉伸“长板”推动岳阳发展优势优先，通过补齐“短板”推动岳阳发展跨越赶超。

加快构建现代产业新格局，着力提高发展质量。坚持以工业经济先行突破带动市域经济整体提升，实施新开工项目“2+10”工程，力争通过三年努力，打造2家投资过百亿、产值过两百亿、税收过十亿元的领军企业，10家投资过十亿、产值过二十亿、税收过亿元的标杆企业。坚定不移引项目，做实园区板块。严格落实产业园区专业化特色化发展实施方案，引导各园区重点发展2—3个主导产业，推动同一园区项目同质集聚，避免不同园区产业同构竞争。继续推行产业项目引进和竣工“双考核”，深入开展“产业招商月”和“引老乡、回故乡、建家乡”活动，聚焦3类“500强”企业及行业龙头企业精准招商，确保经开区、新港区、绿色化工产业园每年分别引进和竣工投资过10亿元工业项目1个以上，其他工业园区每年分别引进和竣工投资过5亿元工业项目1个以上，形成产业招商“头雁效应”。继续抓好省定“5个100”重大产业项目和市定“双百工程”建设，加快己内酰胺搬迁扩能升级、新金宝年产1300万台喷墨打印机、和润粮油、东龙科技、复星合力新材料、龙智新材料、远大可建、卓尔诺科技等重大产业项目建设，以大项目推动园区大发展。加强工业地产管理，实施“135”工程升级版，形成产业项目定向落户、批量落地的“加速度”。坚定不移调结构，做优产业体系。强化科技创新，推动城陵矶新港区晋升为国家级高新区，加快6906军民融合北斗应用产业园、哈工大机器人（岳阳）军民融合创新中心、湖南科伦研究院、东方雨虹等重大科技创新和重大产品创新项目建设。推动制造业高质量发展，提质改造石油化工、食品加工等传统产业，培育壮大装备制造、生物医药、电子信息等新兴产业，加快推进“个转企”“小升规”，助推传统产业“老树发新芽”，新兴产业“小苗成大树”，特别注重挖掘电磁设备制造“隐形冠军”企业潜力，加快打造全国最大电磁产业基地。强化人才支撑，深入实施巴陵人才工程，坚持招商引资与招才引智双管齐下、万众创新与大众创业双轮并进，建立健全以需求为导向、以企业为主体的产学研一体化创新机制，打造岳阳“双创”升级版。坚定不移抓环境，做强民营经济。我们将全面落实习近平总书记在民营企业座谈会上的重要讲话精神、省里关于促进民营经济高质量发展“25条”举措和我市服务企业优化环境“九条措施”，坚持供给侧结构性改革不动摇，在“巩固、增强、提升、畅通”八个字上下功夫，为民营经济发展保驾护航。优化政务环境，深化“最多跑一次”改革，锁定对标台州、全省一流、群众满意的目标，大力推行一件事一窗办、工程建设项目审批联合办、政务服务事项网上办、服务下沉就近办、群众投诉马上办；优化金融环境，严格落实普惠性、结构性减税降费政策，用足用好政府性融资担保公司、小微企业信贷风险补偿基金和各类产业发展基金，建立金融信用信息平台，对符合授信条件但暂时经营困难的企业不抽贷、不压贷、不断贷，依托金融政策加压加力撬动非公企业和中小企业发展提质提效；优化法治环境，维护企业合法权益，以硬措施优化软环境，以软环境提升硬实力，真心成就企业家，真诚厚待投资者，真情服务纳税人，让企业更有预期、更有尊严。

加快构建开放带动新格局，着力放大发展优势。深入推进开放崛起战略，力争全市集装箱吞吐量突破70万标箱、进出口总额突破40亿美元，奋力争当湖南开放崛起的“排头兵”。建设大港口。建成运营新港二期，加快综保区二期建设，积极申报进境水果指定口岸，拓展完善平台功能。依托长江黄金岸线，培育引进一批强链补链型、涉水亲水型企业，加快形成前有高效率水港、后有高质量工厂空间布局；按照“南客北货”战略定位，谋划布局航空货运物流、电子元器件等产业，积极培育空港经济增长点，努力构建水港、空港“双港驱动”，新港区、综保区、空港经济区“三区联动”大开放格局。打通大通道。推进全国多式联运示范工程建设，突出抓好城陵矶高速、松杨湖铁路专用线建设，谋划推进华容煤炭铁水联运储配基地铁路专用线、南洋洲铁路专用线建设，尽快构建成环成网的多式联运集疏运体系。做强大物流。落实“千亿物流产业行动方案”，做大做强临港汽车城物流园、海吉星农产品物流园、中粮物流产业园、胥家桥综合物流园等物流园区，引进培育一批物流龙头企业，大力发展第三方、第四方物流，加快打造国家级物流枢纽城市。发展大旅游。突出抓好洞庭湖博物馆、屈子文化园二期、天岳幕阜山国际度假旅游区、洋沙湖小镇二期等景区景点建设，着力打造古城文化游、山水休闲游、乡村度假游、民俗风情游四大板块；大力实施“旅游+”战略，培育发展城旅、文旅、农旅等新业态，推动旅游由“门票经济”向“产业经济”转变；强化旅游联盟区域合作，促进线路互联、客源互送、市场互享；加快推进刘山庙旅游服务设施、洞庭湖旅游母港、洞庭文体小镇、友阿国际广场等配套设施，东方铂尔曼大酒店等国际五星级酒店和首批“SUC可持续发展国际示范社区”建设，努力打造全域旅游目的地城市。开放带来进步，封闭必然落后，我们将乘着改革开放40周年的东风，扬帆奋进，勇毅前行，敞开大门促发展，让开放的岳阳走出国门、走进深蓝、走向世界！

加快构建城乡统筹新格局，着力拓展发展空间。抓好顶层设计，建立健全双向对流、互补均衡的新型城乡关系。突出融城提质，大力推进国家区域性中心城市建设。着力构建“1+4+2”大岳阳城市圈，提高岳阳大城市的带动力和首位度。加大融城力度。推动区划融城，加快岳荣（岳阳—荣家湾）融城步伐，拓展城市空间；推动产业融城，完善新港区、经开区等各工业园区生产生活配套设施，为产城融合作铺垫、打基础；推动交通融城，加快城市环线和进出通道建设，拉开城市骨架。加大提质力度。坚持治理与开发相结合，强力推进东风湖新区建设，打造城市宜居“名片”；坚持新城建设与旧城改造相结合，加快高铁新城、空港经济区建设，加强历史文化名城保护，抓好洞庭南路历史街区保护性开发与改造，留住城市成长“年轮”；坚持城市修补与生态修复相结合，加大禁拆治违管控、背街小巷改造和城市“疮疤”整治力度，严格执行《岳阳市城市规划区山体水体保护条例》，坚决做到不填湖、不挖山、不毁绿，保护城市生态“基因”。突出示范带动，大力推进乡村振兴战略。打造规范村民建房岳阳样板，推动村庄规划全覆盖，确保竣工集中建房点47个，统筹抓好农村“厕所革命”和“大棚房”问题清理整治，努力补齐影响农村生活品质的短板；升级“空心房”整治岳阳模式，推动“空心房”整治向大面延伸，带动复垦复绿，改善农村人居环境；探索农业产业化特色小镇创建岳阳路径，到2020年成功创建10个特色小镇，打响“特色小镇看岳阳”地域品牌；完善农村移风易俗岳阳机制，推动文明乡风、良好家风、淳朴民风蔚然成风。突出产业强县，大力推进县域经济发展。坚持统筹施策、分类指导，做强中心城区城市经济发展“火车头”，构建临长地区长岳经济走廊“共同体”，建设沿江环湖地区绿色发展“示范区”，整体提升县域经济实力。建设长岳经济走廊，谋划推进长岳城际铁路、长汨大道和虞公港等项目前期工作，大力发展省会“卫星城”经济、工业“飞地经济”和旅游“后花园”经济，敞开岳阳的“南大门”。用心帮扶中小企业发展，着力解决企业融资难、用地难、用工难等现实难题，真正做到“扶上马、送一程”，培育壮大一批专精特新“小巨人”企业，挺起县域经济发展的“脊梁”。加快发展现代农业，坚持藏粮于地、藏粮于技，巩固提升粮食产能，合理调整“粮经饲”结构，增加优质绿色农产品供给；鼓励工商资本下乡，加快农村土地流转，培育发展农业新型经营主体，推动农业从增产导向向提质导向转变。繁荣发展现代服务业，大力发展金融服务、智慧物流、研发设计等生产性服务业，文旅休闲、健康养老、网络信息等生活性服务业，培育更多县域经济增长点。突出城乡一体，大力推进城乡基础设施建设。重点抓好防洪保安工程、中小河流治理、水库除险加固等10类水利设施，洞庭湖北部地区补水工程、大通湖东垸分洪闸建设、长江崩岸治理等9大市级重点工程建设，新增高效节水灌溉面积2.5万亩；加快平江抽水蓄能电站、华电平江电厂、岳阳君山LNG接收站（储备中心）等项目建设，启动实施电网提质改造三年行动计划，开工110千伏及以上电网项目16个；坚持因地制宜、分类施策、集中与分散相结合，统筹抓好乡镇污水处理设施建设，推动城乡基础设施联网对接、共建共享。

加快构建立体交通新格局，着力夯实发展基础。坚持把交通建设作为基础中的基础，放大岳阳区位交通优势。全力打造四通八达的铁路网。抓紧编制《岳阳地区铁路总图规划》，依托蒙华铁路建成通车，畅通岳阳与西北、华东地区大宗货物运输；谋划推进常岳九铁路建设，健全疏港铁路运输体系；推动开通“中欧快线”国际货运班列，把岳阳建设成为中部地区“一带一路”重要节点城市。全力打造覆盖城乡的公路网。加快平益高速公路、G353岳阳东站至土马段建设，打通市域快速通道；加快望岳路北延线、求索西路延伸段等8条城市道路建设，建成运营城市北环线和郭镇公交基地，提升城区道路通行能力；加快自然村通水泥（沥青）路和窄路加宽、危桥改造、安防工程建设，努力解决群众出行难问题。全力打造通江达海的水路网。编制实施《岳阳港总体规划》，加强长江、湘江干线岳阳段疏浚治理，提高船舶通行能力；整合港口岸线资源，构建以城陵矶港区为枢纽港、各县市区港区为喂给港的一体化运营新格局，促进港口集约高效发展；推动港澳直航常态运营，让长江“黄金水道”产生“黄金效益”。全力打造便捷高效的航空网。完善三荷机场配套设施，开通岳阳至北京、上海、成都、西安等10条航线，形成“多米字型”航线网络，尽快建成运营岳阳航空货运枢纽。全力打造智能发达的信息网。扎实推进通信基础设施能力提升三年行动计划，着力建设千兆示范城市和千兆光网小区；加快政务信息化工程建设，打破信息孤岛；加快5G商用步伐，加强人工智能、工业互联网、物联网等新型基础设施建设，以大数据带动数字经济大发展。

加快构建生态发展新格局，着力坚守发展底线。坚持以创建国家长江经济带绿色发展示范区为总揽，持续提高岳阳绿色发展“含金量”，推动经济效益、社会效益、生态效益同步提升。坚持生态优先，守护好一江碧水。强力推进环保督察反馈问题整改，统筹抓好工业园区排污、黑臭水体、矮围网围整治和畜禽水产养殖污染防治、欧美黑杨清退、河湖“清四乱”等工作，巩固提升环保督察成果；严格落实河湖长制，突出抓好长江、湘江、洞庭湖等大江大湖治理，努力把长江岸线打造成为“最美岸线”，把洞庭湖区打造成为“大美湖区”；切实抓好东洞庭湖国家级自然保护区生态保护与修复、铁山库区水源地保护与民生保障工作，坚决查处非法养殖、非法捕捞、违规建房等行为，守护好生态功能保护区休养生息、万物和谐的自然风光；加强与三峡集团合作，加快污水处理厂提标改造和污水管网建设，扎实抓好城乡污水处理收费改革试点和省级海绵城市创建工作，充分凸显岳阳城水相依、人水相亲的宜居魅力；系统推进大气污染和土壤污染防治，坚决打赢蓝天、碧水、净土三大“保卫战”。坚持绿色发展，转变发展方式。增加绿色生产供给，加强“化工围江”整治，引导化工企业向绿色化工产业园集聚，加强工业园区绿色化循环化改造，严格年耗能1万吨标煤以上重点企业节能审查监管，加快淘汰落后产能，大力发展绿色循环经济；提高资源利用效率，合理确定建设用地开发强度，清理闲置低效用地，推进“腾笼换鸟”；倡导低碳生活方式，大力推广氢能公交和新能源汽车，鼓励低碳建筑、环保建材、节能家电等绿色消费，努力建设节约型社会。坚守生态保护红线、环境质量底线和资源利用上线，是新时代赋予我们的重大使命，必须以万全准备确保万无一失，用最大努力争取最好结果，真正让岳阳人民身边的湖水清澈如镜、头顶的天空湛蓝如画、脚下的大地翠绿如茵！

四、顺应新时代美好愿景，共建共享全面小康幸福生活

人民群众对美好生活的向往，就是我们的奋斗目标。2019年是全面建成小康社会的关键之年，我们要锁定目标，全面对标跟进，让岳阳发展每一项成果真正惠及全市人民。

精准精细推进脱贫攻坚。行百里者半九十，脱贫攻坚已进入了倒计时，必须牢牢托住“两不愁三保障”扶贫工作底线，密切关注脱贫新动态，确保全年脱贫2万人以上。坚持精准扶贫、精准脱贫基本方略。严格落实到村到户到人精准帮扶“十大举措”，统筹推进就业扶贫“311”工程、“百企帮百村”行动、八大种养产业帮扶行动和补齐贫困地区基础设施短板“四大行动”，完善稳定脱贫长效机制，不断提高贫困地区和贫困人口自我发展能力。坚持重点推进、全域推进有机结合。对非贫困村与贫困村的贫困人口脱贫工作同步安排、同步推进，注重解决非贫困村“插花式”贫困问题；探索城市扶贫新模式，注重解决城市特困家庭、特困群体最低生活保障问题；保持现行脱贫标准不变，注重防范和解决贫困群众陷入“福利陷阱”、对非贫困人口造成“悬崖效应”的问题；加大对收入水平略高于建档立卡贫困户群体的政策支持力度，注重解决贫困“夹心层”问题。坚持强化责任、强化监管两手发力。压实党政主导、干部主帮、乡镇主推、农户主体的扶贫责任体系，构建专项扶贫、行业扶贫、社会扶贫互为补充的大扶贫格局；加强扶贫领域作风建设，严格扶贫资金检查、审计和监督，确保扶贫资金使用“滴水不漏”。到2020年，实现现行标准下农村贫困人口全部脱贫，坚决做到脱真贫、真脱贫、不返贫。

稳扎稳打防控风险隐患。重大风险隐患防控是一项“100－1=0”的工作，一着不慎，满盘皆输。我们将认真处理好稳增长、促改革与防风险的关系，继续打好防范化解重大风险攻坚战。防控政府债务风险。严格落实隐性债务化解方案，坚决完成年度化债任务；加强政府投资项目全过程管控，停缓调撤一批非必要性、非紧迫性、非公共性政府投资项目，严控债务增量；加强国有资产管理，实施国有资产清理整治专项行动，推动平台公司转型发展，努力实现从管企业向管资本转变，增强国有资产造血功能和平台公司偿债能力；推进棚改征拆清零攻坚，加快处置存量土地，加速资金回笼，降低项目成本；严格预算绩效管理，建立“花钱必问效、无效必问责”硬性约束机制，牢牢守住政府债务风险“警戒线”。统筹抓好稳金融工作，坚决守住金融风险“总闸门”。防控安全生产风险。坚持党政同责、一岗双责、失职追责，深入开展安全生产隐患大排查大管控大整治行动，加强道路交通、危化物品、公共场所等重点领域安全监管，进一步夯实基层基础，坚决杜绝重特大安全生产事故发生。防控社会稳定风险。加大“平安岳阳”创建力度，深入开展扫黑除恶专项斗争，严厉打击黑恶势力犯罪及其“保护伞”，持续推进“三无”村（社区）创建活动，大力实施“雪亮工程”，提高社会治安综合治理智能化水平；深入开展打击电信网络违法犯罪专项行动，整顿规范“网络贷”市场，坚决打击套路贷、校园贷、高利贷等金融诈骗行为，严格查处非法集资行为；严肃整治房地产市场建筑质量、违法抵押、延期交房、物业管理等矛盾集中凸显的问题，维护购房者合法权益；加强自然灾害、事故灾难、公共卫生、食品药品、社会安全等应急管理，确保突发事件矛盾不激化、不升级；加强信访法治化建设，抓好信访“三无”单位创建工作。通过高强度管控“风险点”，筑牢高质量发展“防火墙”，着力营造更加稳定、更加宽松、更加和谐的社会环境。

加速加力繁荣社会事业。坚持教育优先，稳步推进城乡义务教育一体化和高考改革，加大城东片区、学院路片区、湖滨片区、城北片区中小学校布局调整力度，完成中小学校“六建六扩”任务，统筹抓好高等教育、职业教育和民办教育，促进各类教育均衡发展，努力办好人民满意教育。推进“健康岳阳”战略，突出抓好市疾控中心迁建、市儿童医院新建、市一医院东院二期、市二医院门诊大楼、市中医院基本医疗住院大楼等项目建设，加快县级医院综合能力建设和基层卫生院、卫生室标准化建设，抓好无偿献血工作，保障医疗用血安全。深入开展全民健身运动，加快第十四届省运动会场馆建设。实施文化惠民工程，全面改扩建工人文化宫，确保市图书馆新馆投入使用，积极做好国家卫生城市复审迎检工作，巩固提升国家公共文化服务体系示范区和全国文明城市创建成果。扎实做好民族宗教工作。加快推进企业“三供一业”社会职能移交，减轻国有企业办社会的压力。切实抓好统计改革和第四次全国经济普查、第七次全国人口普查。坚持依法治网管网，加强网络舆情引导和处置，让更多的正能量主导传递“岳阳声音”。

全心全意办好民生实事。坚持把以人民为中心作为政府工作价值取向，努力把经济总量、财政增量转化为老百姓实实在在的生活质量。全面完成省定实事任务，重点办好10件市级民生实事：一是完成棚改项目38个，改善7926户棚户区居民住房条件；二是加强中心城区交通秩序综合整治，新建公共停车场4处，完成人行过街设施建设3处，建成投运智能交通项目；三是中心城区升级置换公交候车亭262个，更换纯电动新能源公交车200台；四是完成公路生命安全防护工程1300公里；五是实现“一村一辅警”全覆盖，建成“城市快警”平台20个；六是标准化建设改造农贸市场13家；七是完成新生儿48种遗传代谢病串联质谱筛查4.5万人；八是帮助1600名处于就业年龄段的残疾人享受居家和集中托养服务；九是建设义务教育标准化教学点45个、农村公办幼儿园15所；十是提质改造湖滨、临港、南津港3处污水处理厂，完成中心城区东风湖等5处黑臭水体整治。全面增进民生福祉，群众热切期盼，政府义不容辞，我们将一件一件办好民生实事，一条一条兑现民生承诺，一个一个破解民生难题，确保全市人民更多地共享政策红利、共享发展成果！

五、担当新时代职责使命，努力建设人民满意政府

加强政府自身建设，是做好政府工作的重要保障。我们将认真抓好中央、省委巡视组反馈问题整改，努力造就忠诚、干净、担当的高素质干部队伍，加快建设人民满意政府。

突出政治建设，让思想“多补钙”。政府班子既要埋头干事，又要抬头看路。我们将把政治建设摆在首位，自觉坚持“两个维护”，落实“三个坚决”，增强“四个意识”，对党中央的大政方针、战略部署和重大决策，在思想上坚决对标对表，在行动上坚决跟紧跟进，在任务上坚决落地落细，始终保持对党绝对忠诚，牢牢站稳政治立场。

突出法治建设，让权力“进笼子”。政府权力既要守住边界，又要管住弹性。我们将大力推进依法行政，严格执行《岳阳市政府工作规则》，全面实施“三张施政清单”，坚决落实行政执法“三项制度”，切实规范行政自由裁量权，主动接受人大、政协监督和社会监督，通过标定“边界线”，划定“硬杠杠”，确保行政权力在法治的轨道上、在监督的阳光下健康运行。

突出效能建设，让改革“出动能”。政府履职既要讲求规范，又要追求效能。我们将全面深化改革，完成机构改革任务，统筹抓好经济体制改革、社会体制改革、农村综合改革等各项改革，加快破除不利于经济高质量发展、不利于社会公平性发展、不利于城乡普惠性发展的体制机制障碍，让改革充分释放岳阳发展永不枯竭的动能。

突出作风建设，让队伍“增活力”。政府工作既要真抓实干，又要真心容错。我们将力戒文山会海，力兴调研之风，力破形式主义，力推干在一线，让一线工作法成为作风建设的主旋律。坚决执行中央、省里关于进一步激励广大干部新时代新担当新作为的有关文件精神，严格落实“三个区分开来”，宽容干部失误，关心干部成长，为干事者撑腰，为担当者担当，激励广大干部干事创业、建功立业。

突出廉政建设，让行为“守规矩”。政府形象既要守住底线，又要拔高标线。我们将把群众满意作为最高标准，担硬党风廉政建设主体责任，严格落实“一岗双责”，严格执行中央“八项规定”及实施细则精神，严格抓好公共资源交易等六大领域“提篮子”行为专项整治，通过正身正行、正人正己的“本色出演”，不断提高政府公信力，让政府系统清正在心、清流涌动、清风长在。

各位代表，浪急催潮涌，风好正扬帆！新时代的宏伟序幕已经拉开，新时代的嘹亮号角已经吹响。让我们紧密团结在以习近平同志为核心的党中央周围，在中共岳阳市委的坚强领导下，万众一心绘好同心圆，协力同心筑梦新时代，奋力谱写建设湖南发展新增长极的宏伟篇章，以无愧于时代的发展业绩、无愧于人民的工作实绩，向新中国成立70周年献礼！
